# Exploratory machine-learning analysis of bevacizumab exposure and circulating VEGF dynamics in patients with cancer

**DOI:** 10.3389/fphar.2026.1899527

**Published:** 2026-10-09

**Authors:** Ileana Theofili, Elias Liolis, Angelos Koutras, Vangelis D. Karalis, Gregory Sivolapenko

**Affiliations:** 1 Department of Pharmacy, National and Kapodistrian University of Athens, Athens, Greece; 2 Department of Medicine, University of Patras, Patras, Greece; 3 Department of Pharmacy, University of Patras, Patras, Greece

**Keywords:** bevacizumab, cancer, VEGF dynamics, machine learning

## Abstract

**Introduction:**

This study explored pharmacokinetic and pharmacodynamic factors associated with circulating vascular endothelial growth factor (VEGF) dynamics during bevacizumab treatment.

**Methods:**

A secondary analysis was conducted using data from 74 patients with breast, ovarian, cervical, non-small cell lung, or colorectal cancer. Paired pre- and post-infusion serum concentrations of bevacizumab and VEGF were measured by ELISA. Exploratory dimensionality-reduction methods and supervised tree-based models were applied. Artificial neural networks were used only as a complementary exploratory analysis on the same dataset.

**Results:**

The main outcome of the analysis was VEGF_D, the absolute difference between the paired VEGF concentrations. The supervised models did not perform above the naive three-class benchmark; therefore, their variable-importance outputs were treated as secondary, model-specific, and hypothesis-generating observations. The results show exploratory model patterns of bevacizumab exposure and circulating VEGF dynamics. We did not assess clinical efficacy, safety results, and other dosing strategies in this study.

**Discussion:**

The results do not support clinical implementation, personalized dosing, or treatment optimization and need to be confirmed in large independent cohorts.

## 1 Introduction

Recent advances in cancer biology have established sustained proliferative signaling, cell death resistance, and angiogenesis as key components of cancer progression (Hanahan and Weinberg, 2011). By making new blood vessels, tumors obtain oxygen and nutrients and develop pathways for metastatic spread (Garcia et al., 2020; Carmeliet, 2005; Lugano et al., 2020). The dependence of many solid tumors on angiogenic signaling has therefore provided a rationale for therapies targeting the vascular endothelial growth factor (VEGF) pathway, including tyrosine-kinase inhibitors and monoclonal antibodies (Figure 1) (Papachristos et al., 2020; Liu et al., 2023). However, tumor vascular phenotypes and angiogenic programs vary substantially among and within cancer types (Lugano et al., 2020).

**FIGURE 1:**
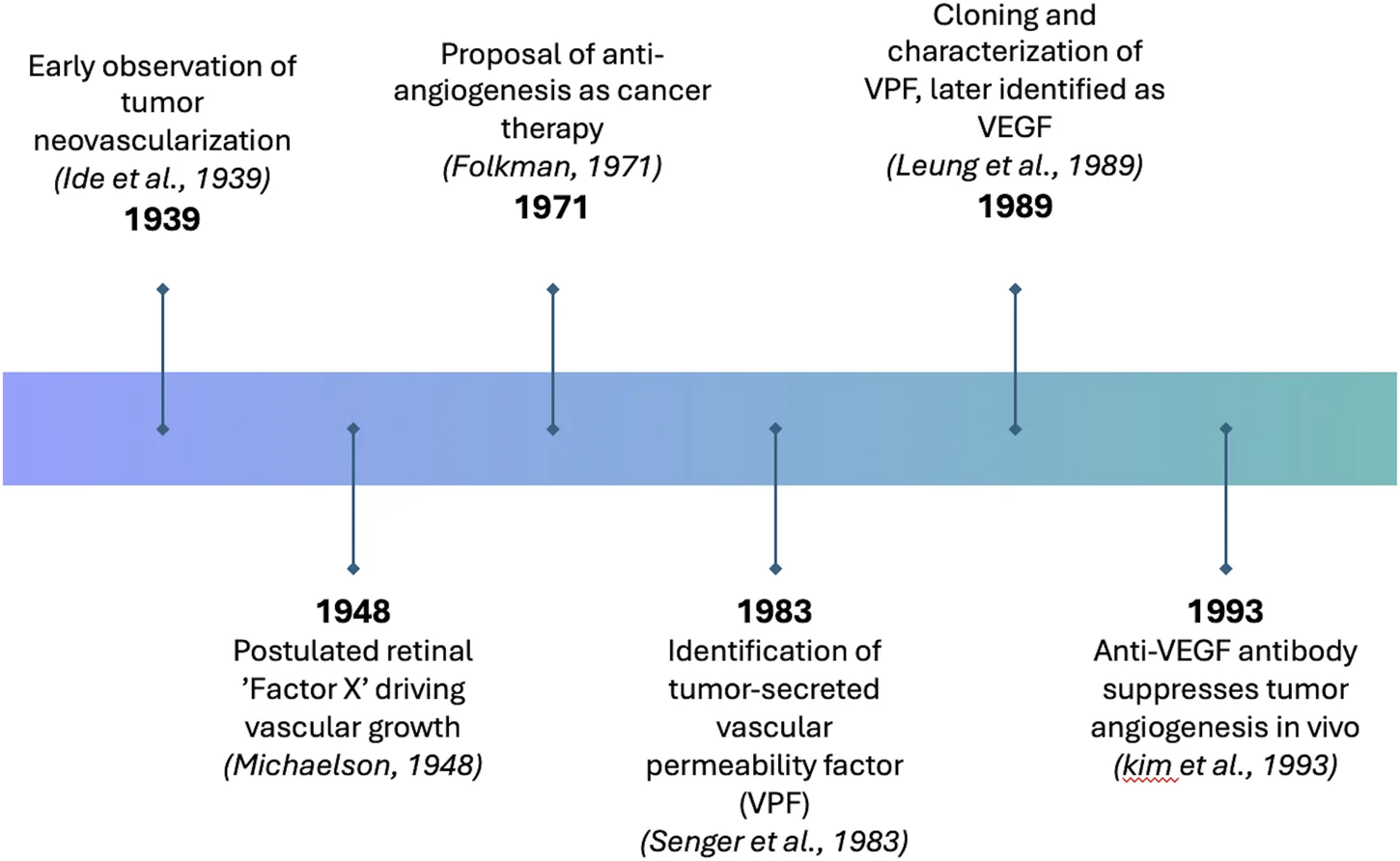
Timeline of key milestones in the discovery of vascular endothelial growth factor (VEGF) and tumor angiogenesis (Ide et al., 1939; Michaelson, 1948; Folkman, 1971; Senger et al., 1983; Leung et al., 1989; Kim et al., 1993).

Tumor angiogenesis reflects an imbalance between pro- and anti-angiogenic signals (Elayat and Selim, 2024). The VEGF family includes VEGF-A, VEGF-B, VEGF-C, VEGF-D, and placental growth factor (PlGF). VEGF-A and VEGF-B primarily support angiogenic signaling, whereas VEGF-C and VEGF-D contribute prominently to lymphangiogenesis (Melincovici et al., 2018; Ohishi et al., 2023). These ligands signal through VEGFR-1, VEGFR-2, and VEGFR-3 (Ahmad and Nawaz, 2022). VEGF-A, which exists in several splice isoforms, is a major mediator of pathological angiogenesis and vascular permeability and acts principally through VEGFR-1 and VEGFR-2 on endothelial cells. Hypoxia-inducible factor 1-alpha (HIF-1α) and alterations affecting the von Hippel–Lindau (VHL) pathway can increase VEGF-A expression (Ahmad and Nawaz, 2022; Albini et al., 2024). Bevacizumab is a humanized monoclonal antibody that binds VEGF-A and inhibits its interaction with VEGFR-1 and VEGFR-2 (Figure 2) (Ferrara et al., 2004).

**FIGURE 2:**
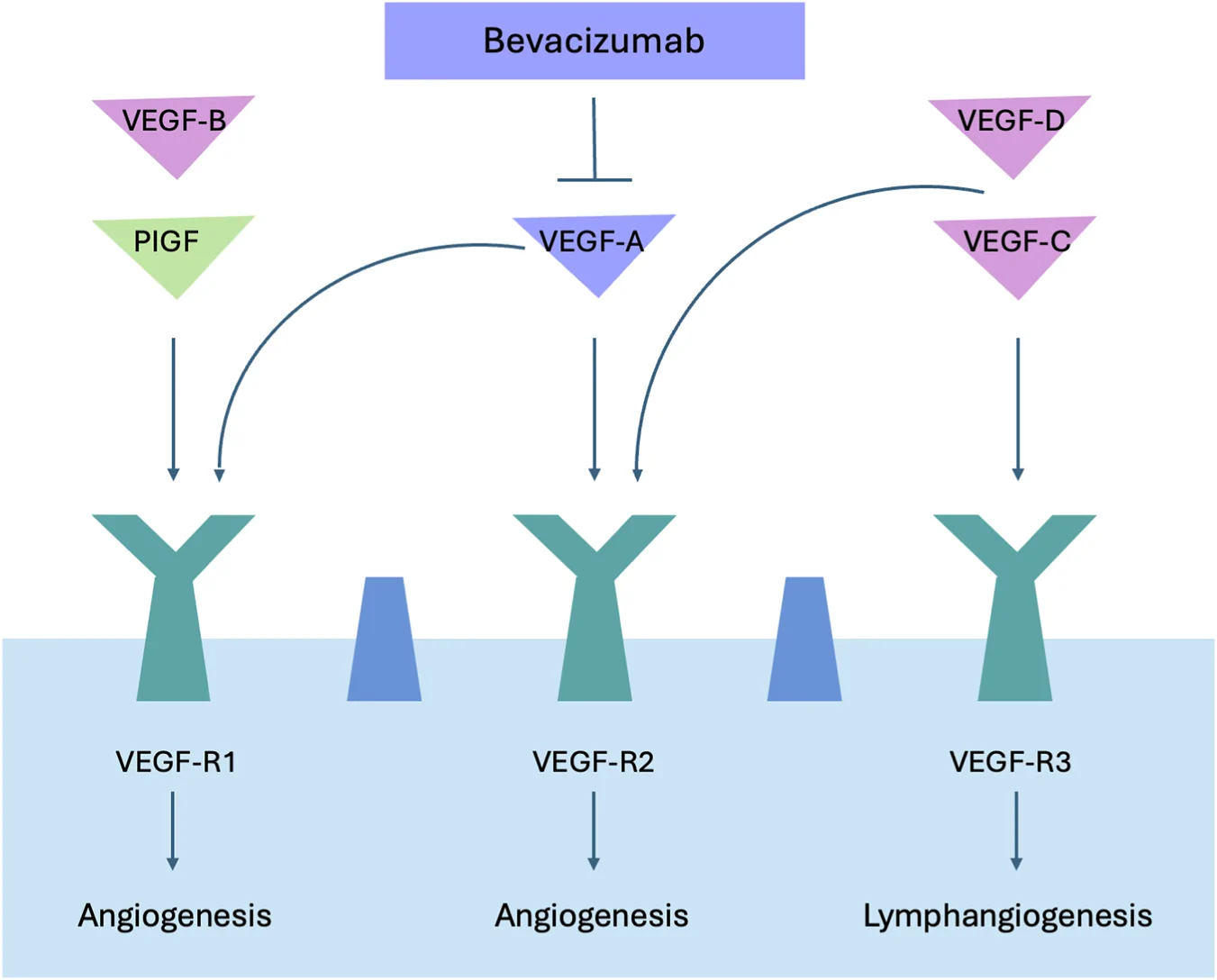
Schematic representation of bevacizumab inhibition of vascular endothelial growth factor A (VEGF-A) signaling. VEGF-A binds VEGF receptor 1 (VEGFR-1) and VEGF receptor 2 (VEGFR-2), with VEGF-A–VEGFR-2 signaling primarily involved in angiogenesis. Placental growth factor (PlGF) and VEGF-B bind VEGFR-1, whereas VEGF-C and VEGF-D bind VEGF receptor 3 (VEGFR-3) and contribute to lymphangiogenesis. Bevacizumab binds VEGF-A and inhibits its interaction with VEGFR-1 and VEGFR-2.

Bevacizumab is a recombinant humanized monoclonal antibody and was the first anti-angiogenic agent approved for cancer therapy, first in metastatic colorectal cancer (Gerber and Ferrara, 2005; Ferrara et al., 2004). It is currently approved in combination with other cancer therapies in colorectal, non-small cell lung, renal, cervical, ovarian, fallopian-tube, and primary peritoneal cancer. Bevacizumab is also approved for metastatic breast cancer in combination regimens (European Medicines Agency, 2026). Depending on the indication, bevacizumab is administered with chemotherapy and may be continued as maintenance therapy. The magnitude and type of clinical benefit vary by disease setting and regimen and should not be generalized across all approved indications (Coleman et al., 2017; Rossi et al., 2017).

Machine learning (ML) is a subfield of artificial intelligence that helps in data-driven pattern recognition, prediction and hypothesis generation using supervised, unsupervised and reinforcement learning methods (Karalis, 2024b). Deep learning is the type of ML based on multilayer neural networks; convolutional neural networks (CNNs), recurrent neural networks and transformers are used primarily for large, high-dimensional data and can reduce the need for manual feature design (Karalis, 2024a; LeCun et al., 2015). In this work, PCA, CATPCA and FAMD were used for exploratory dimension reduction, Random Forest, gradient-boosted trees and bagged trees were used as tree-based ML models, and ANN was a shallow feed-forward neural-network classifier (not deep learning). In this context, for this study, we explored patterns among demographic characteristics, treatment variables, bevacizumab concentrations, and circulating VEGF in patients with non-small cell lung, colorectal, breast, cervical, or ovarian cancer. The study was not designed to predict clinical outcomes, optimize dosing, or to support personalized treatment.

## 2 Methods

### 2.1 Study design

This secondary analysis used anonymized real-world data from a prospective observational clinical study conducted at the Department of Oncology, University General Hospital of Patras ‘Panagia Voithia,’ Patras, Greece. The original study was conducted in accordance with the Declaration of Helsinki and International Council for Harmonisation Good Clinical Practice and was approved by the Ethics Committee of the University General Hospital of Patras ‘Panagia Voithia’ (protocol number 5377/35,833; approval date 24 November 2017). Before enrollment in the original study, all participants provided written informed consent. The original informed consent form authorized the future research use and secondary analysis of participants’ anonymized data. The present secondary analysis was therefore conducted within the scope of the original ethics approval and informed consent. No additional informed consent was required for this analysis, and no waiver of the original written informed consent was applied. No new patient contact, intervention, or sample collection occurred.

A total of seventy-four (74) patients with different types of cancer were studied (breast cancer, n = 23; ovarian cancer, n = 27; cervical cancer, n = 7; non-small cell lung cancer, n = 7; colorectal cancer, n = 10). All patients were between 33 and 78 years old, with a median age of 60 years. The cohort included eight male patients (10.8%) and 66 female patients (89.2%) (Table 1).

**TABLE 1:** Baseline demographic, clinical, and concentration summaries of the study population.

Characteristic	Total patients (N = 74, 100.0%)	Breast cancer (N = 23, 31.1%)	Ovarian cancer (N = 27, 36.5%)	Cervical cancer (N = 7, 9.5%)	NSCLC (N = 7, 9.5%)	CRC (N = 10, 13.5%)
Gender	​	​	​	​	​	​
Male	8 (10.8%)	3 (13.0%)	0 (0.0%)	0 (0.0%)	3 (42.9%)	2 (20.0%)
Female	66 (89.2%)	20 (87.0%)	27 (100.0%)	7 (100.0%)	4 (57.1%)	8 (80.0%)
Age (median, range)	60 (33–78)	59 (43–78)	60 (33–78)	60 (48–76)	60 (39–68)	61 (48–75)
Types of therapy	​	​	​	​	​	​
Monotherapy	23 (31.1%)	11 (47.8%)	7 (25.9%)	3 (42.9%)	2 (28.6%)	0 (0.0%)
Combination	51 (68.9%)	12 (52.2%)	20 (74.1%)	4 (57.1%)	5 (71.4%)	10 (100.0%)
Bevacizumab concentrations, median (range)	​	​	​	​	​	​
Bevacizumab trough (AV_T; μg/mL)	141.3 (0.0–756.3)	224.1 (0.0–544.8)	177.9 (0.0–593.7)	143.2 (0.0–756.3)	29.5 (0.0–535.0)	28.7 (0.0–141.3)
Bevacizumab peak (AV_P; μg/mL)	665.0 (56.6–1,535.0)	722.7 (267.4–1,487.4)	707.4 (350.2–1,535.0)	762.2 (56.6–1,378.6)	533.9 (201.0–1,014.8)	314.0 (61.0–771.4)
Absolute difference between paired bevacizumab measurements (AV_D; μg/mL)	494.6 (38.4–1,135.6)	509.5 (123.5–1,080.0)	515.9 (164.9–1,080.6)	524.0 (56.6–1,135.6)	488.6 (38.4–800.0)	293.8 (61.0–767.7)
Assay-detectable serum VEGF-A concentrations, median (range)	​	​	​	​	​	​
Post-infusion VEGF-A (VEGF_T; pg/mL)	128.5 (0.0–979.0)	86.0 (0.0–665.7)	116.3 (0.0–979.0)	25.1 (0.0–377.7)	53.4 (0.0–431.0)	280.4 (36.8–705.1)
Pre-infusion VEGF-A (VEGF_P; pg/mL)	512.2 (4.2–1,310.0)	406.0 (28.0–1,097.1)	533.4 (30.2–1,310.0)	439.4 (4.2–982.0)	452.2 (5.2–1,040.0)	831.8 (290.5–1,261.4)
Absolute difference between paired VEGF-A measurements (VEGF_D; pg/mL)	357.1 (28.0–925.7)	311.7 (28.0–863.3)	369.6 (30.2–925.7)	301.7 (49.2–697.7)	328.1 (39.4–854.3)	472.8 (110.3–825.8)

All patients received bevacizumab (Avastin, Roche) according to standard clinical practice and the applicable marketing authorization. Twenty-three patients received bevacizumab monotherapy, and 51 received bevacizumab in combination with chemotherapy. Treatment exposure ranged up to 23 cycles. Also, all patients received first-line bevacizumab-containing treatment. Patients with colorectal cancer (CRC) received bevacizumab by intravenous infusion at a dose of 5 mg/kg every 2 weeks in combination with 5-fluorouracil/leucovorin/irinotecan or oxaliplatin (BEV-FOLFIRI or BEV-FOLFOX, respectively). Patients with non-small cell lung cancer (NSCLC), breast cancer, or gynecologic malignancies received bevacizumab at a dose of 15 mg/kg by intravenous infusion every 3 weeks in combination with carboplatin and paclitaxel for up to six cycles. Treatment was initially administered for up to six cycles, and in patients who responded favorably, therapy was continued with bevacizumab-based maintenance treatment.

### 2.2 Bioanalysis

Two serum samples were collected during each evaluated treatment cycle. The first sample was obtained 1 h before bevacizumab infusion and was designated as the bevacizumab trough (AV_T) and corresponding pre-infusion VEGF measurement (VEGF_P). The second sample was obtained immediately after completion of the infusion and was designated as the bevacizumab peak (AV_P) and corresponding post-infusion VEGF measurement (VEGF_T). The bevacizumab peak/trough labels refer operationally to the two protocol-defined sampling times rather than to a complete concentration–time profile. Bevacizumab concentrations were quantified using a direct ELISA (Creative Diagnostics, USA). VEGF-A was quantified using the Quantikine Human VEGF immunoassay (R&D Systems, USA), a sandwich ELISA designed to detect natural and recombinant human VEGF-A, including VEGF165 and VEGF121. Because bevacizumab may interfere with VEGF capture and detection and the assay may detect dissociated or complexed VEGF, the measurements are referred to throughout this manuscript as assay-detectable serum VEGF-A. They should not be interpreted as validated measurements of total VEGF-A or exclusively free (unbound) VEGF-A (Sumner et al., 2019). Bevacizumab concentrations are reported in μg/mL, whereas assay-detectable serum VEGF-A concentrations are reported in pg/mL. Throughout the manuscript and figures, VEGF_P denotes the pre-infusion VEGF measurement and VEGF_T denotes the post-infusion VEGF measurement; peak/trough terminology is reserved for bevacizumab.

Sampling and derived-variable sensitivity analyses. The pre-infusion sample was collected 1 h before bevacizumab administration and defined AV_T and VEGF_P, whereas the post-infusion sample was collected immediately after completion of the infusion and defined AV_P and VEGF_T. The bevacizumab peak/trough labels are operational definitions for the two protocol-specified sampling times and are not indicative of the concentration-time profile as a whole. AV_D was calculated as |AV_P − AV_T| and VEGF_D as |VEGF_P − VEGF_T|. Sensitivity outcomes included the individual pre-infusion VEGF_P and post-infusion VEGF_T measurements, signed percentage VEGF reduction [100 × (VEGF_P − VEGF_T)/VEGF_P], and the post/pre VEGF ratio (VEGF_T/VEGF_P). To evaluate whether AV_D contributed information beyond either individual bevacizumab measurement, two prespecified incremental models were fitted: AV_D was added to an adjusted model containing AV_P, and separately to an adjusted model containing AV_T. Because AV_D = AV_P − AV_T for all patient-level records, AV_D, AV_P, and AV_T were not entered simultaneously as three separate predictors, which would create exact algebraic collinearity. Models were adjusted for body weight, treatment duration, age, sex, cancer type, and treatment regimen and used HC3 heteroscedasticity-consistent standard errors. The corresponding incremental and alternative-outcome analyses are summarized in Supplementary Table S2.

Following blood collection, samples were transferred into serum collection vials and centrifuged at 1,500 × g for 10 min at 4 °C to separate the serum. The serum fraction was carefully aspirated using a pipette and transferred into Eppendorf tubes, which were stored at −20 °C until further analysis. Prior to analysis, samples were removed from the freezer and allowed to equilibrate to room temperature. They were then diluted using the appropriate dilution buffers supplied with the ELISA kits. Defined volumes of the diluted samples were dispensed into the ELISA plates using automatic pipettes, and all subsequent steps were performed according to the manufacturer’s instructions.

Bevacizumab serum concentrations were measured using a direct ELISA method, in which antigens present in the patient serum bind to pre-coated antibodies on the plate, followed by the addition of an enzyme-conjugated secondary antibody. Upon substrate addition, a colorimetric reaction occurred only if the enzyme was bound to the antigen–antibody complex, with color intensity directly proportional to the bevacizumab concentration. VEGF quantification was performed using a sandwich ELISA. In this assay, a capture antibody immobilized on the plate first bound VEGF present in the serum, followed by the addition of a detection antibody supplied in the kit. An enzyme-linked reagent was subsequently added, producing a colorimetric signal proportional to the VEGF concentration. Absorbance was measured using a microplate reader (ThermoMax, Molecular Devices, San Diego, CA, USA) at 450/470 nm for the direct ELISA and 450/630 nm for the sandwich ELISA. Each kit included a standard curve with a corresponding linear regression equation (y = ax + b), where y represents absorbance and x the analyte concentration. Sample concentrations were calculated using this equation and adjusted for the dilution factor to obtain the final serum concentrations of bevacizumab and VEGF.

The present study was designed as an exploratory pharmacokinetic/pharmacodynamic secondary analysis. VEGF_D, defined as the absolute change between the paired pre- and post-infusion serum VEGF measurements, was used as the primary analytical outcome to characterize circulating VEGF dynamics. Clinical efficacy and safety outcomes, including objective tumor response, progression-free survival, overall survival, and treatment-related toxicity, were not available and were not analyzed. Consequently, VEGF_D was not treated as a validated surrogate endpoint for clinical benefit. Accordingly, AV_D was evaluated exclusively as an exposure-related predictor of circulating VEGF_D within this exploratory PK/PD framework. AV_D was not evaluated against clinical endpoints, and no association between AV_D and treatment efficacy or patient benefit can be inferred from the present analysis.

### 2.3 Machine learning analysis

All analyses were conducted in Python. The exploratory dimension-reduction methods, supervised models, preprocessing steps, software versions, model specifications, and validation procedures are described in the final reproducibility workflow below.

PCA is a widely used ML method that reduces dimensionality while preserving as much information as possible. This method transforms the original data space into a new one that represents a linear combination of the original variables. PCA is applied when the dataset contains only numerical variables. The resulting dimensions are called principal components (PCs), each of which explains a portion of the variance in the dataset. The contribution of each original variable to a PC is referred to as a “loading,” and values closer to one (positive or negative) indicate a stronger positive or negative influence on the corresponding PC (Jolliffe and Cadima, 2016). CATPCA is an exploratory dimension-reduction method for categorical or ordinal inputs. It produces a lower-dimensional representation of the input matrix while retaining as much variation as possible. In this study, vector positions, angles, and lengths were interpreted descriptively and were not used as formal tests of association, independence, or biological influence (Infantosi et al., 2014). Factor analysis of mixed data (FAMD) is an exploratory dimension-reduction method for datasets containing both continuous and categorical variables. It combines PCA-based treatment of standardized continuous variables with multiple-correspondence-analysis treatment of categorical variables so that neither data type dominates the solution. In this study, FAMD plots were used descriptively to visualize patterns among mixed variables and were not interpreted as formal tests of association or independence (Sayadi et al., 2021).

The reproducibility workflow used for the final analysis was as follows. The analysis used one row per patient (n = 74). When a patient had more than one analytical record, AV_T, AV_P, VEGF_T, and VEGF_P were averaged at the patient level before AV_D = |AV_P − AV_T| and VEGF_D = |VEGF_P − VEGF_T| were calculated. For PCA, the continuous variables shown in each figure panel (AV_P, AV_T, VEGF_P, VEGF_T and, where indicated, Cycles, Weight, AV_D, and VEGF_D) were z-standardized. CATPCA was operationalized by converting continuous inputs to cohort tertiles coded 1–3 and applying PCA to the standardized ordinal matrix. FAMD used Age, Weight, Cycles, Duration, AV_T, AV_P, AV_D, VEGF_T, VEGF_P, and VEGF_D as continuous variables and Sex, Cancer_Type, and Therapy as categorical variables; continuous variables were standardized and categorical variables were internally indicator-encoded. For exploratory supervised classification analyses, we grouped VEGF_D into cohort-specific tertiles to obtain three approximately balanced outcome classes: low (≤308.03 pg/mL), middle (>308.03 to ≤452.58 pg/mL), and high (>452.58 pg/mL), comprising 25, 24, and 25 patients, respectively. The predictors were AV_D (μg/mL), Weight (kg), and Cycles. This classification was used only to develop models and is not a clinically valid threshold. Since categorization may lead to loss of information, we have retained VEGF_D as a continuous outcome for the correlation and regression analyses. There were no missing values in these predictors or the target; nevertheless, a median imputer fitted only to each training partition was retained in the pipeline and did not replace any values. Tree models used unscaled predictors, whereas ANN inputs were standardized using training-set means and standard deviations. A stratified 85:15 split produced 62 training and 12 test patients, with random seed 42. Five-fold stratified cross-validation with shuffling and the same seed was performed within the training set; hyperparameters were prespecified and were not tuned. The Random Forest used 500 trees, square-root feature sampling, a minimum leaf size of 2, and balanced class weights. Gradient boosting used 100 trees, a learning rate of 0.05, a maximum depth of 2, and a minimum leaf size of 2. Bagging used 500 bootstrap trees, 80% of training samples per tree, and a minimum leaf size of 2. The ANN was a feed-forward classifier with three input nodes, one hidden layer of eight ReLU units, and a three-node softmax output layer; it used the Adam optimizer, a learning rate of 0.001, a batch size of 8, and a maximum of 1,000 epochs. Early stopping used 20% of the training fold as validation data, a tolerance of 10–4, and patience of 50 epochs. Accuracy, macro-precision, macro-recall, and macro-F1 were calculated for the held-out test set and cross-validation. Analyses were conducted in Python 3.12.13 using pandas 2.2.3, NumPy 2.3.5, scikit-learn 1.8.0, and prince for FAMD; plots were generated with Matplotlib. The corresponding held-out and cross-validation metrics are summarized in Supplementary Table S1. Because the three outcome classes were nearly balanced, an accuracy of approximately 0.333 for a naive three-class classifier was used as a descriptive benchmark. AV_D, body weight, and treatment cycles were prespecified predictors and were not selected through an unrestricted feature-selection procedure.

Variable-importance calculation. The variable importance was calculated from the models fit to the training data. For the Random Forest and gradient boosted trees, the importance was calculated by the normalized total reduction in the splitting criterion due to each predictor. For the bagged tree, the predictor importance was averaged over the base trees and normalized over the three predictors. For the ANN, the relative predictor importance was calculated using the absolute products of the input-to-hidden and hidden-to-output connection weights, summed over the hidden units and output classes and normalized over the three predictors. For all models, the importance scores were normalized to sum to one across the three predictors. The held-out test set was used only to assess predictive performance and not to determine variable importance or select predictors.

Handling of repeated treatment-cycle records: The selected cohort comprised 89 paired pre- and post-infusion analytical records from 74 patients: 59 patients contributed one paired record and 15 contributed two paired records obtained during different treatment cycles. Because the study’s inferential and predictive unit was the patient, repeated records were aggregated before model fitting. This strategy retained all available concentration measurements while preventing the 15 patients with two sampled cycles from receiving twice the analytical weight of patients with one sampled cycle. AV_T, AV_P, VEGF_T, and VEGF_P were averaged within patient before AV_D and VEGF_D were calculated. Consequently, every primary statistical and machine-learning analysis used one row per patient (n = 74), and no patient could occur in more than one data partition or cross-validation fold.

Repeated-record sensitivity analysis: To verify that aggregation did not mask an association and to account explicitly for the correlation between records from the same patient, the adjusted AV_D–VEGF_D regression was repeated using all 89 cycle-level paired records. The model included AV_D per 100 μg/mL and the same prespecified covariates as the primary model: body weight, treatment duration, age, sex, cancer type, and treatment regimen. Inference used a CR1 sandwich covariance estimator clustered by PatientID, including the finite-sample correction, with two-sided t tests and 95% confidence intervals based on 73 cluster degrees of freedom. Thus, this sensitivity analysis retained the repeated cycle-level observations while explicitly accounting for within-patient correlation.

In addition to the exploratory dimension reduction plot, we conducted formal statistical analyses to assess the relationship between AV_D and VEGF_D. The main unadjusted analysis used Spearman’s rank correlation as the difference measures were not expected to be normally distributed. We calculated the two-sided p-value and 95% percentile bootstrap confidence interval of 20,000 patient-level resamples (random seed 42) of the study. The Pearson correlation coefficient, together with a Fisher z-transformed 95% confidence interval, was examined as a sensitivity analysis. A multivariable ordinary least-squares model was developed with continuous VEGF_D (pg/mL) as the outcome and AV_D (per 100 μg/mL) as exposure of interest. The model was adjusted *a priori* for body weight (per 10 kg), treatment duration, age (per 10 years), sex, cancer type (indicator variables; breast cancer as reference), and treatment regimen (combination therapy versus monotherapy). Heteroscedasticity-consistent HC3 standard errors and two-sided P values were used. All 74 patients had complete data for these variables, and statistical significance was defined as P < 0.05. These formal tests, rather than PCA/CATPCA vector geometry, were used for inferential interpretation. The corresponding formal association estimates are summarized in Supplementary Table S2.

Interpretation of demographic and treatment covariates. Sex, cancer type, and treatment regimen were retained as adjustment covariates but were not evaluated as confirmatory subgroup hypotheses. The cohort contained eight men and 66 women; cancer-type strata ranged from 7 to 27 patients; and 23 patients received monotherapy compared with 51 receiving combination therapy. Cancer type, dosing interval, and treatment regimen were also partly aligned by clinical indication. Given these sparse, unequal, and interrelated groups, estimates for these variables were interpreted descriptively; failure to detect a model signal was not treated as evidence of equivalence, independence, or absence of association.

## 3 Results

### 3.1 Principal component analysis

Interpretive framework for PCA and CATPCA. PCA and CATPCA were used exclusively as exploratory visualization and dimensionality-reduction tools. Vector direction, angle, and length were treated as geometric features of reduced-dimensional projections and not as hypothesis tests of association, independence, or biological influence. Accordingly, statements based on vector orientation were interpreted descriptively and were not used as primary evidence for the AV_D–VEGF_D relationship.

Exploratory PCA (Figure 3A) showed close alignment within the paired VEGF variables (VEGF_P and VEGF_T) and within the paired bevacizumab variables (AV_P and AV_T). The two variable groups were separated in the two-dimensional projection. The first two components explained 76.86% of the total variance (40.30% and 36.56%, respectively). These geometric patterns are reported descriptively and were not interpreted as formal evidence of association or independence.

**FIGURE 3:**
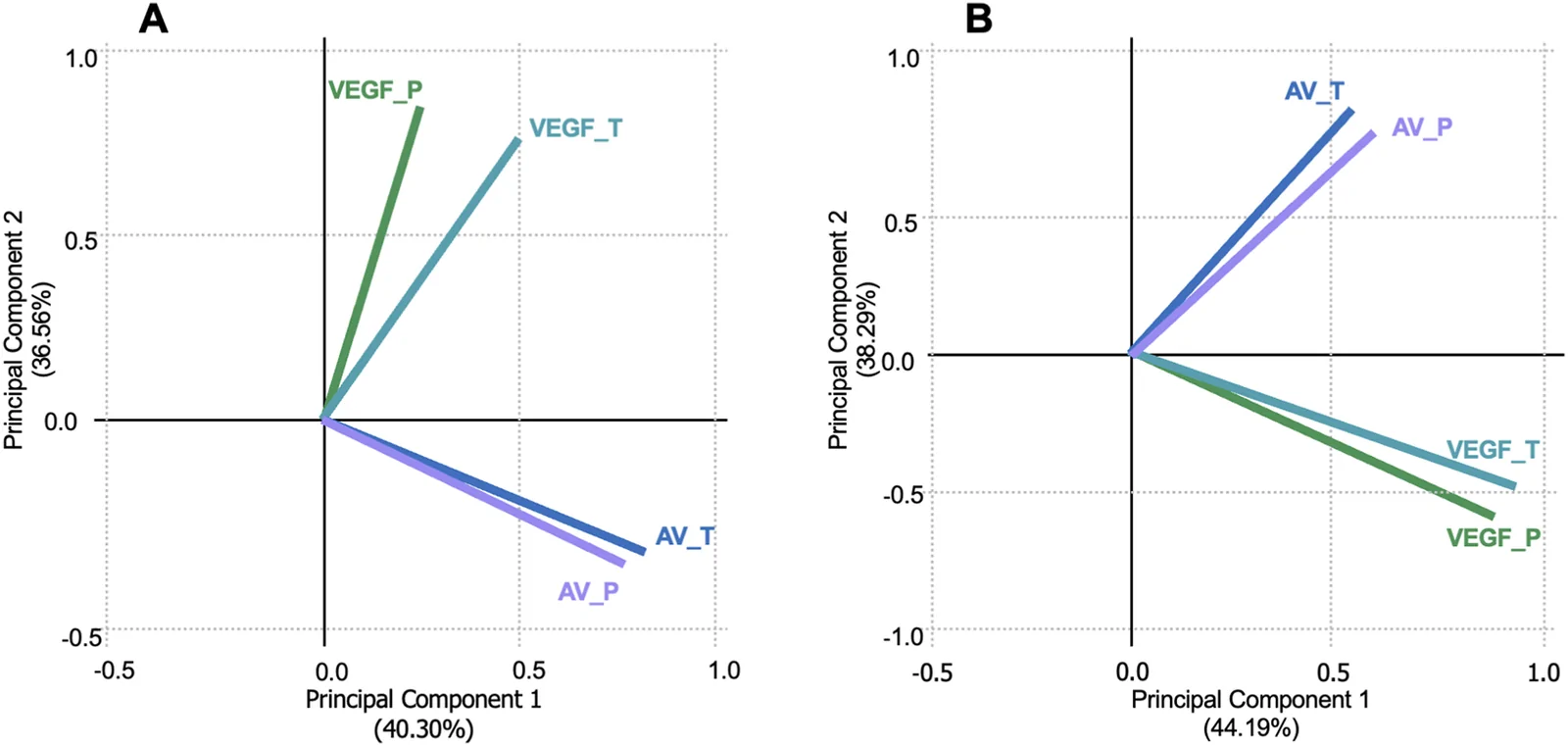
Exploratory principal component analysis and categorical principal component analysis (CATPCA) visualizations of paired bevacizumab and vascular endothelial growth factor (VEGF) measurements. **(A)** PCA projection of bevacizumab peak (AV_P), bevacizumab trough (AV_T), pre-infusion VEGF (VEGF_P), and post-infusion VEGF (VEGF_T). The accompanying table presents component loadings for the first two dimensions. **(B)** CATPCA projection of AV_P, AV_T, VEGF_P, and VEGF_T. Vector orientation and category positions are descriptive features of reduced-dimensional projections and do not constitute formal statistical evidence of association or independence.

CATPCA (Figure 3B) explained 82.48% of the variance, of which 44.19% was due to the first dimension and 38.29% to the second. VEGF_P and VEGF_T were aligned to one another, as were AV_T and AV_P, while the VEGF and bevacizumab groups remained separated in the projection. Since CATPCA has been used as an exploratory visualization method, the vector geometry is not used to determine when, how, and by what biological mechanism the VEGF response occurs.

Exploratory PCA and CATPCA (Figure 4) showed close alignment between treatment duration or cycle number and bevacizumab concentrations, particularly AV_P, whereas the VEGF vectors were less closely aligned with the treatment variables. The first two PCA components explained 64.66% of the total variance, and the first two CATPCA dimensions explained 78.39%. These patterns describe the reduced-dimensional projections only and do not establish drug accumulation or a causal effect of treatment duration. Body weight was included in a series of exploratory PCA projections with bevacizumab and VEGF variables (Figure 5). These projections were used to describe the relative positions of the variables under different combinations and were not treated as formal tests of association or biological influence.

**FIGURE 4:**
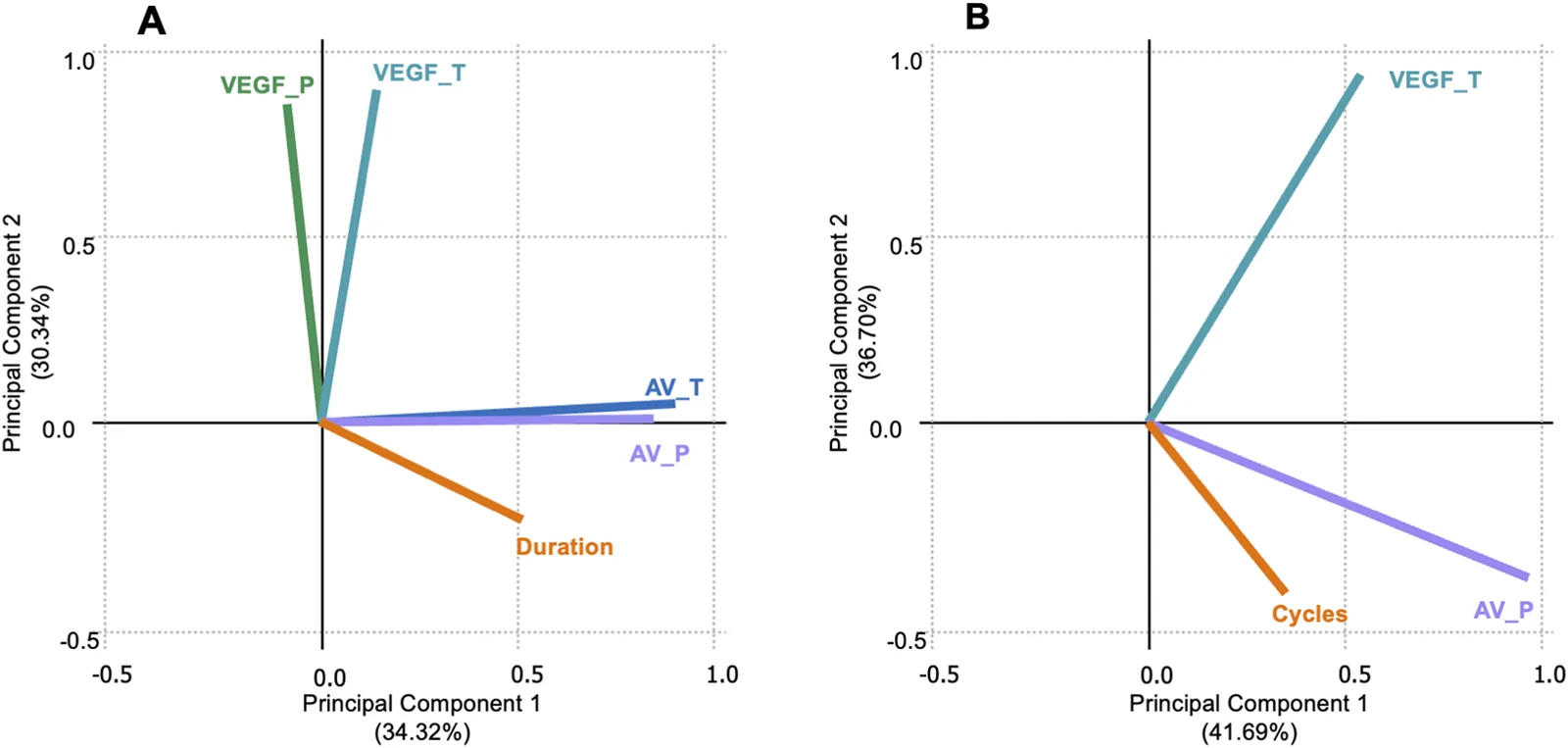
Exploratory principal component analysis and categorical principal component analysis (CATPCA) images of treatment duration and paired bevacizumab and vascular endothelial growth factor (VEGF) measurements in cycles. **(A)** PCA projection of bevacizumab peak (AV_P), bevacizumab trough (AV_T), pre-infusion VEGF (VEGF_P), post-infusion VEGF (VEGF_T) and treatment cycles. **(B)** CATPCA projection of AV_P, VEGF_T and treatment cycles. The plots are exploratory, and the relative direction and category positions should not be considered as tests of association, independence or biological influence.

**FIGURE 5:**
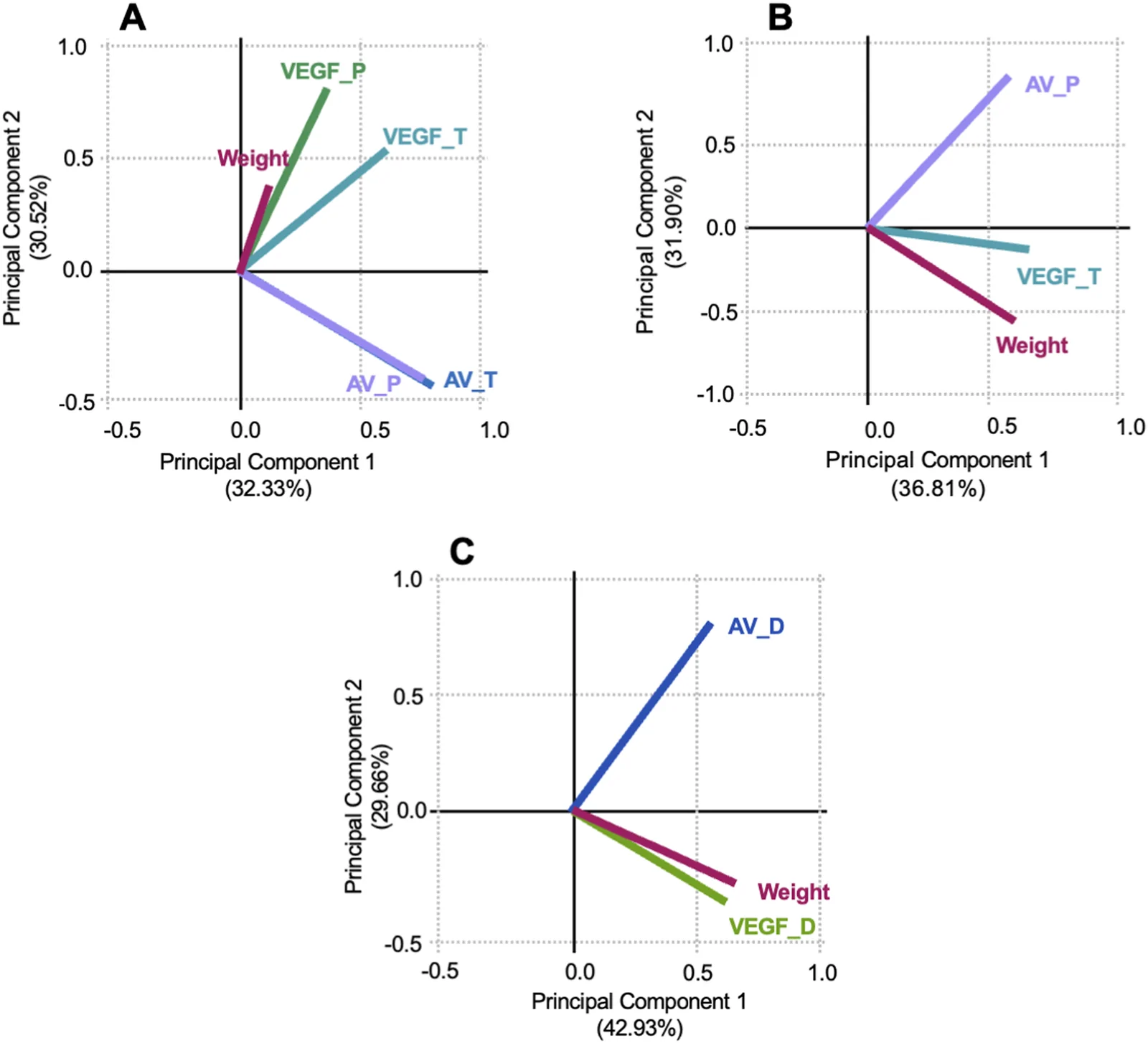
Exploratory principal component analysis visualizations of body weight and bevacizumab/vascular endothelial growth factor (VEGF) measurements. **(A)** Projection of bevacizumab peak (AV_P), bevacizumab trough (AV_T), pre-infusion VEGF (VEGF_P), post-infusion VEGF (VEGF_T), and body weight. **(B)** Projection of AV_P, VEGF_T, and body weight. **(C)** Projection of body weight, the absolute difference between paired bevacizumab measurements (AV_D), and the absolute difference between paired VEGF measurements (VEGF_D). Vector alignment is presented descriptively and does not constitute formal statistical evidence of association or biological influence.

We noticed in exploratory PCA models of body weight (Figure 5) that weight was close to the VEGF variables in the two-dimensional projections and the bevacizumab variables were more distant. The first two components accounted for 62.85%, 68.71%, and 72.05% of the variance in panels A, B, and C. In panel C, weight and VEGF_D are close together, but AV_D is far apart. These are descriptive patterns and do not imply any correlation, causality, or biological activity.

Formal analyses provided a more detailed inferential assessment of the relationship between AV_D and VEGF_D. The Spearman correlation was ρ = −0.017 (95% CI −0.257 to 0.229; P = 0.886), and the Pearson sensitivity analysis was concordant (r = −0.017, 95% CI −0.245 to 0.212; P = 0.885). In the multivariable model adjusted for body weight, treatment duration, age, sex, cancer type, and treatment regimen, the AV_D coefficient was −4.93 pg/mL in VEGF_D per 100 μg/mL increase in AV_D (95% CI −30.05 to 20.18; P = 0.696). These formal estimates distinguish conventional linear or monotonic association from the nonlinear, model-specific information summarized by exploratory projections and variable-importance rankings. These formal association estimates are summarized in Supplementary Table S2.

The repeated-record sensitivity analysis was consistent with the primary patient-level inference. Using all 89 cycle-level paired records and patient-clustered robust standard errors, the adjusted AV_D coefficient was −1.73 pg/mL in VEGF_D per 100 μg/mL increase in AV_D (95% CI −22.71 to 19.24; P = 0.870). This concordance indicates that the inferential conclusion was not dependent on the patient-level aggregation strategy. The result is included in Supplementary Table S2.

The sensitivity analyses were concordant across exposure parameterizations and VEGF outcome definitions. After adjustment for body weight, treatment duration, age, sex, cancer type, and treatment regimen, adding AV_D to the model containing AV_P changed R^2^ by 0.0009; the AV_D coefficient was 5.71 pg/mL in VEGF_D per 100 μg/mL increase in AV_D (95% CI −47.43 to 58.85; P = 0.831). Adding AV_D to the corresponding model containing AV_T changed R^2^ by 0.0030; the coefficient was −6.18 pg/mL (95% CI −30.44 to 18.09; P = 0.613). In adjusted analyses using alternative outcomes, the AV_D coefficients per 100 μg/mL were −8.07 pg/mL for VEGF_P (95% CI −41.53 to 25.39; P = 0.631), −22.36 pg/mL for VEGF_T (95% CI −62.07 to 17.35; P = 0.265), 2.56 percentage points for signed percentage VEGF reduction (95% CI −3.02 to 8.13; P = 0.363), and −0.0256 for the post/pre VEGF ratio (95% CI −0.0813 to 0.0302; P = 0.363). The fact that the estimates are similar across other endpoints suggests that the interpretation does not depend on a single outcome of VEGF. The sensitivity analysis results are presented in Supplementary Table S2.

We also explored the relevance of demographic and treatment covariates. In these exploratory analyses, sex, cancer type, and treatment regimen were not prominent predictors in this cohort. The absence of a detectable signal under the present analytical conditions should not be interpreted as evidence that these variables are unrelated to VEGF. The small number of men, unequal cancer-type groups, and imbalance between monotherapy and combination therapy limited the precision and power of subgroup comparisons; therefore, no claim of equivalence or biological independence was made.

### 3.2 Ensemble methods of analysis

As a secondary exploratory analysis, supervised tree-based models were used to examine whether AV_D, body weight, and treatment cycles contained information for classifying tertile-defined VEGF_D. Model performance was evaluated before relative variable importance was interpreted. Because performance did not exceed the naive three-class benchmark, the importance outputs were retained only as descriptive, model-specific rankings within this dataset and not as evidence of association, effect, or predictive value.

The Random Forest model ranked AV_D as the most important, then body weight and treatment cycles. As the model did not perform above the naive three-class benchmark, this order is only descriptive of the fitted model in our dataset and does not imply that the variables are associated with, influenced by or predict VEGF_D. The corresponding variable-importance plot is provided in Supplementary Table S1.

Supervised model performance was quantified using complementary held-out and cross-validation metrics. In the held-out test set, accuracy, macro-precision, macro-recall, and macro-F1 were, respectively, 0.250, 0.306, 0.250, and 0.261 for Random Forest; 0.333, 0.361, 0.333, and 0.328 for boosted trees; 0.333, 0.512, 0.333, and 0.338 for bagged trees; and 0.333, 0.229, 0.333, and 0.269 for the ANN. Mean five-fold cross-validation accuracy and macro-F1 within the training set were 0.240 and 0.223 for Random Forest, 0.272 and 0.262 for boosted trees, 0.256 and 0.233 for bagged trees, and 0.306 and 0.282 for the ANN. Because the three VEGF_D classes were nearly balanced, the expected accuracy of a naive three-class classifier was approximately 0.333. Held-out accuracies ranged from 0.250 to 0.333, and mean five-fold cross-validation accuracies ranged from 0.240 to 0.306. Thus, none of the supervised models demonstrated predictive performance above the naive reference benchmark. The importance scores represent only a relative ranking of the three prespecified predictors within the fitted models. Although AV_D was generally ranked first, followed by body weight and treatment cycles, this ordering is model-specific and dataset-dependent. It may be used only for hypothesis generation and does not demonstrate predictive utility, biological relevance, or an independent association with VEGF_D. Detailed model-performance results are presented in Supplementary Table S1.

The boosted- and bagged-tree models produced a similar descriptive ordering of the three prespecified predictors, with AV_D ranked first, followed by body weight and treatment cycles. Because these models were fitted to the same dataset and did not perform above the naive reference benchmark, similarity in their rankings does not constitute independent validation or evidence of robustness. The rankings are retained solely as model-specific, dataset-dependent observations for hypothesis generation and should not be interpreted as evidence of association, effect, biological relevance, or predictive value. The corresponding variable-importance plots are provided in Supplementary Table S2.

### 3.3 Artificial neural networks

Finally, an ANN was applied as a complementary exploratory analysis using the same dataset and predictor set as the tree-based models. Given the small sample size (n = 74) and the absence of an independent validation cohort, the ANN output was used only to describe model-specific variable-importance rankings.

In the ANN analysis, AV_D received the highest relative importance ranking, followed by body weight and treatment duration. A similar ordering was observed in the tree-based models. However, because all models were fitted to the same cohort and showed chance-level predictive performance, this similarity is descriptive only and does not provide evidence of association, biological relevance, predictive validity, or independent validation. The corresponding variable-importance plot is provided in Supplementary Table S3.

## 4 Discussion

The present study was designed as an exploratory pharmacokinetic/pharmacodynamic analysis of bevacizumab exposure variables and circulating VEGF dynamics. It did not assess treatment efficacy, safety, or other dosing strategies. Circulating VEGF is only a portion of the VEGF biology associated with bevacizumab treatment and cannot capture spatially heterogeneous intratumoral signaling (Lambrechts et al., 2013; Caulet et al., 2016). Our analytical approach was therefore used to generate hypotheses about patterns in PK/PD data in the absence of clinical outcome data. The exploratory dimension-reduction and variable-importance analyses identified descriptive patterns involving treatment duration, body weight, and VEGF_D. These patterns should not be interpreted as evidence of bevacizumab accumulation, an effect of body weight, a biological mechanism, or independence from treatment regimen. The rankings and formal estimates are presented descriptively, and any mechanistic explanations should be regarded only as hypotheses requiring further evaluation.

Given the chance-level performance of the supervised models, the observed variable-importance rankings provide no evidence of predictive validity or biological relevance. They are retained only as model-specific, dataset-dependent observations that may support hypothesis generation for evaluation in larger independent cohorts.

The additional sensitivity analyses strengthen interpretive clarity by separating the individual bevacizumab concentrations from their derived contrast and by evaluating several complementary VEGF outcomes. AV_D remains a useful compact descriptor of the within-cycle bevacizumab concentration contrast for exploratory nonlinear modeling; however, because it is algebraically determined by AV_P and AV_T, it should not be interpreted as an independent source of information beyond both component concentrations. The concordant results for VEGF_P, VEGF_T, signed percentage reduction, and the post/pre ratio support a calibrated, outcome-robust interpretation of the exploratory findings.

The demographic and treatment-regimen findings warrant particular caution. With only eight men, cancer-specific groups as small as seven patients, and unequal regimen groups, weak or subgroup-specific effects could remain undetected. In addition, cancer type, dosing interval, and treatment regimen were partly linked by clinical indication, limiting attribution to any single factor. Accordingly, the present analysis does not establish that VEGF dynamics are unaffected by sex, cancer type, or treatment regimen; it indicates only that these variables were not prominent predictors in this exploratory pooled cohort. Confirmation requires larger, balanced, disease-specific cohorts with prespecified subgroup analyses.

Consistent circulating VEGF levels have been reported to be associated with disease progression or survival in some tumors (Miyazaki et al., 2008; Xu et al., 2023), but studies of bevacizumab-treated patients need to be carefully interpreted. VEGF clearance after bevacizumab has been altered by the drug, and post-treatment increases in circulating VEGF may reflect the host pharmacology and not tumor production (Ferrara et al., 2004; Alidzanovic et al., 2016). Sampling time and anti-VEGF interference with the Quantikine assay can influence the measured values (Sumner et al., 2019). So, the assay-detectable serum levels of VEGF-A in these tumors are only a timing- and assay-dependent systemic biomarker and should not be interpreted as a direct indicator of tumor-specific or exclusively free, biologically active VEGF-A.

Bevacizumab exhibits approximately linear pharmacokinetics over the 0.3–10 mg/kg dose range and reaches steady-state concentrations after repeated administration. Population pharmacokinetic analyses have reported modest sex-related differences in central volume of distribution and clearance; however, these differences are influenced substantially by body weight and body composition (Lu et al., 2008). Lower albumin concentrations and greater tumor burden, both of which can accompany advanced disease, have also been associated with increased bevacizumab clearance. Reported elimination half-lives vary across studies and analytical approaches, including approximately 18 days in women and 20 days in men in two-compartment analyses and a wider range in Bayesian analyses (European Medicines Agency, 2026; Shih and Lindley, 2006; Caulet et al., 2016). These sources of pharmacokinetic variability provide context for heterogeneity in bevacizumab exposure. In the present study, the alignment of treatment duration with bevacizumab concentrations was observed in exploratory PCA/CATPCA projections and was not interpreted as a formally tested association.

AV_D should only be interpreted as the absolute difference between the paired bevacizumab measurements obtained in the study sampling scheme. It should not be interpreted as a measure of sustained exposure, exposure stability, cumulative exposure, or overall drug exposure. Its high variable-importance ranking was model-specific and does not establish a statistical or biological association with VEGF_D, identify a therapeutic target, or indicate that the approved dosing regimens are suboptimal. No alternative dose or administration schedule was evaluated in this study. Previous studies have examined relationships between bevacizumab exposure, circulating VEGF, and clinical outcomes in specific populations (Canonici et al., 2012; Caulet et al., 2016; Pfisterer et al., 2023). These reports provide pharmacological context but cannot be used to infer clinical benefit from the present analysis, because tumor response, progression-free survival, overall survival, toxicity, dose modification, and treatment discontinuation were not analyzed here. Body weight also received a relatively high importance ranking in the exploratory models. Although body size can influence monoclonal-antibody pharmacokinetics (Ternant and Paintaud, 2005; Han et al., 2016), the present results do not demonstrate that approved weight-based dosing is inadequate and do not support an alternative dosing approach.

ANNs can represent nonlinear relationships, but their use in this study was substantially constrained by the sample size of 74 patients. The ANN was trained on the same observations used for the other models and was not evaluated in an external cohort. Its results are therefore complementary and exploratory and cannot be used to validate the tree-based findings or support clinical or dosing conclusions. Future studies should first confirm these exploratory model patterns in larger independent cohorts using prespecified validation procedures. Any assessment of exposure-guided or individualized dosing would additionally require prospective comparison of dosing strategies and evaluation of efficacy and safety endpoints before clinical implementation could be considered.

Several limitations affect interpretation of the modeling results. Circulating VEGF is an indirect systemic measurement and does not represent tumor-specific VEGF signaling. The cohort was small (n = 74), included five tumor types, and was markedly imbalanced by sex, which limits the stability and generalizability of the estimates. All tree-based and ANN models were developed from the same dataset, and no external validation cohort was used. Accordingly, the ANN analysis was complementary and exploratory, not an independent validation step. Similarity in variable-importance rankings across models cannot exclude overfitting or model-dependent artifacts. Confirmation requires larger, more diverse, independent cohorts and a prespecified validation strategy. An additional limitation is the absence of clinical outcome data. Objective tumor response, progression-free survival, overall survival, and treatment-related toxicity were not evaluated. Accordingly, the observed PK/PD patterns cannot be used to assess treatment efficacy, predict patient benefit, or support VEGF_D as a validated surrogate endpoint. The tree-based and neural-network models were developed in a small, heterogeneous cohort of 74 patients and remain susceptible to overfitting. A stratified 85:15 held-out split and five-fold cross-validation within the training set were used to provide complementary performance estimates and prevent preprocessing leakage. However, the held-out test set included only 12 patients, and no external validation cohort was available; therefore, the estimates have limited precision and generalizability. Similar variable-importance rankings across algorithms do not constitute independent validation because all models were fitted to the same cohort. The findings remain exploratory and hypothesis-generating and require confirmation in larger independent cohorts using prespecified validation procedures. The VEGF_D tertile thresholds were derived from this cohort and should not be interpreted as clinically meaningful cutoffs. Their use may reduce information and limit the stability and generalizability of the classification models, particularly given the small sample size.

Pooling patients across five malignancies provided a broader exploratory perspective on bevacizumab PK/PD patterns within a clinically diverse cohort. At the same time, these cancers differ substantially in tumor biology, clinical context, dosing schedules, and concomitant treatments, which limits disease-specific interpretation. Because several subgroups were small, stratified analyses would have produced unstable estimates. The pooled findings, including the variable-importance rankings, should therefore be regarded as context-dependent and hypothesis-generating rather than tumor-independent or broadly generalizable. Nevertheless, this cross-cancer analysis helps identify questions and candidate patterns that can guide more focused evaluation in larger, adequately powered, disease-specific cohorts. Machine-learning methods may be useful for hypothesis generation in pharmacological research. However, the models developed in this small, heterogeneous cohort were not designed or validated as clinical decision-support tools. The present analysis therefore should not be interpreted as enabling precision therapy, real-time monitoring, or personalized anti-VEGF treatment.

Future research should focus on external validation of the model in large, independent, disease-specific cohorts; prespecified model-development procedures with repeated or nested cross-validation; adequately powered analyses of sex, cancer type, dosing interval, and treatment regimen; and longitudinal sampling analyzed with methods that account for within-patient correlation. Future studies should also include efficacy, safety, and treatment exposure endpoints before deciding whether any model-based measure is clinical or dosing-relevant. These are research priorities and not advice for clinical implementation.

## 5 Conclusion

This exploratory study evaluated bevacizumab exposure and circulating VEGF dynamics by combining conventional statistical analyses with complementary machine-learning approaches. This analytical framework characterized patterns within a clinically heterogeneous cohort and identified AV_D, body weight, and treatment duration as features warranting further investigation. The observed variable-importance rankings should be regarded as model-specific and hypothesis-generating and do not establish independent biological associations or predictive value. Nevertheless, the study provides a foundation for future pharmacological research on the relationships among bevacizumab exposure, patient characteristics, and circulating VEGF dynamics. Validation in larger, independent, disease-specific cohorts incorporating relevant clinical endpoints may clarify the biological and potential clinical relevance of these findings.

## Data Availability

The data analyzed in this study is subject to the following licenses/restrictions: The datasets are not publicly available due to ethical and privacy restrictions, as they contain anonymized clinical data derived from cancer patients. Access to the datasets may be considered by the corresponding author upon reasonable request and subject to approval by the relevant ethics and institutional authorities. Requests to access these datasets should be directed to GS, gsivolap@upatras.gr.
