# Supplementary material for: Exploratory machine-learning analysis of bevacizumab exposure and circulating VEGF dynamics in patients with cancer
Source: Front Pharmacol. 2026 Oct 9;17:1899527. doi: 10.3389/fphar.2026.1899527 (PMC13690702; doi:10.3389/fphar.2026.1899527)
Supplement: Supplementary file 1 [file Supplementaryfile1.pdf]

Supplementary Tables

Table S1. Held-out and cross-validated performance of the supervised models.

**Panel A. Held-out test-set performance (n = 12)**				
**Model**	**Accuracy**	**Macro-precision**	**Macro-recall**	**Macro-F1**
Random forest	0.250	0.306	0.250	0.261
Boosted trees	0.333	0.361	0.333	0.328
Bagged trees	0.333	0.512	0.333	0.338
ANN	0.333	0.229	0.333	0.269
**Panel B. Five-fold cross-validation within the training set (n = 62)**				
**Model**	**Accuracy, mean ± SD**	**Precision, mean ± SD**	**Recall, mean ± SD**	**Macro-F1, mean ± SD**
Random forest	0.240 ± 0.123	0.249 ± 0.145	0.237 ± 0.122	0.223 ± 0.119
Boosted trees	0.272 ± 0.072	0.278 ± 0.090	0.270 ± 0.071	0.262 ± 0.078
Bagged trees	0.256 ± 0.091	0.248 ± 0.118	0.253 ± 0.090	0.233 ± 0.093
ANN	0.306 ± 0.094	0.339 ± 0.191	0.313 ± 0.094	0.282 ± 0.111

Note: The held-out test set comprised 12 patients. Five-fold stratified cross-validation was performed within the 62-patient training set. Cross-validation values are mean ± standard deviation. ANN, artificial neural network; SD, standard deviation.

Table S2. Formal association, repeated-record, incremental-exposure, and alternative-outcome analyses.

**Panel A. Primary AV_D–VEGF_D association analyses**				
**Analysis**	**Estimate**	**95% CI**	**P value**	**Scale/adjustment**
Spearman correlation	−0.017	−0.257 to 0.229	0.886	Unadjusted ρ
Pearson correlation	−0.017	−0.245 to 0.212	0.885	Unadjusted r
Adjusted AV_D coefficient	−4.93	−30.05 to 20.18	0.696	pg/mL VEGF_D per 100 µg/mL AV_D
Cycle-level clustered sensitivity	−1.73	−22.71 to 19.24	0.870	89 records; patient-clustered CR1 SE; adjusted
**Panel B. Incremental information from AV_D**				
**Base exposure**	**ΔR²**	**AV_D estimate**	**95% CI**	**P value**
AV_P	0.0009	5.71	−47.43 to 58.85	0.831
AV_T	0.0030	−6.18	−30.44 to 18.09	0.613
**Panel C. Alternative VEGF outcomes**				
**Outcome**	**AV_D estimate**	**95% CI**	**P value**	**Outcome scale**
Pre-infusion VEGF_P	−8.07	−41.53 to 25.39	0.631	pg/mL

Post-infusion VEGF_T	−22.36	−62.07 to 17.35	0.265	pg/mL
Signed percentage VEGF reduction	2.56	−3.02 to 8.13	0.363	percentage points
Post/pre VEGF ratio	−0.0256	−0.0813 to 0.0302	0.363	unitless ratio

Note: Adjusted models included body weight, treatment duration, age, sex, cancer type, and treatment regimen. Primary patient-level models used HC3 heteroscedasticity-consistent standard errors. The cycle-level sensitivity analysis used all 89 records from 74 patients and CR1 standard errors clustered by PatientID with a finite-sample correction. Unless otherwise stated, AV_D coefficients are expressed per 100 µg/mL increase in AV_D. ΔR^2 is the change after adding AV_D to the specified base-exposure model. CI, confidence interval.

Deleted: Percentage

Supplementary Figures

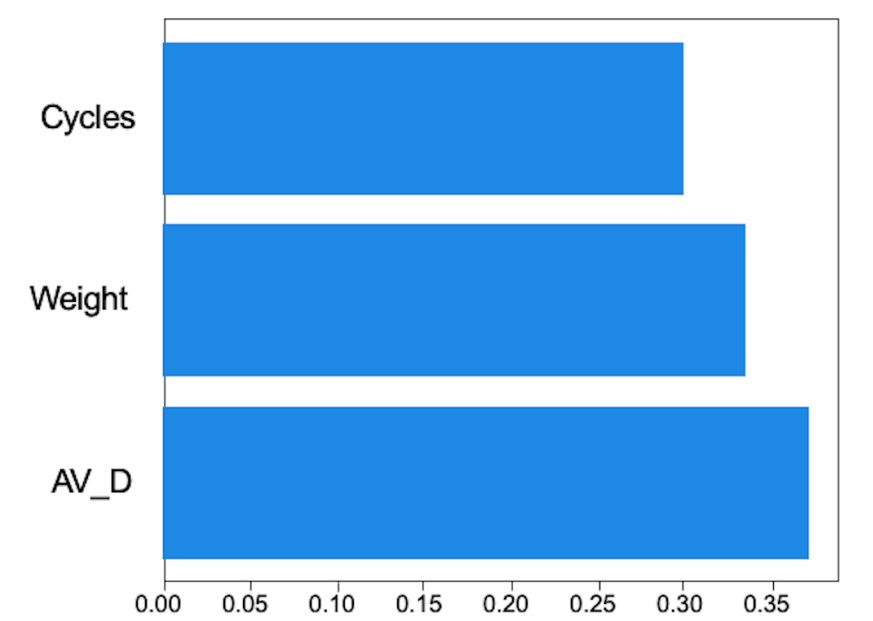

SUPPLEMENTARY FIGURE S1. Random Forest variable-importance scores for the absolute difference between paired bevacizumab measurements (AV_D), body weight, and treatment duration in cycles, with the absolute difference between paired vascular endothelial growth factor measurements (VEGF_D) as the target variable. Importance scores are model-specific descriptive data and cannot be interpreted as P values or evidence of causality.

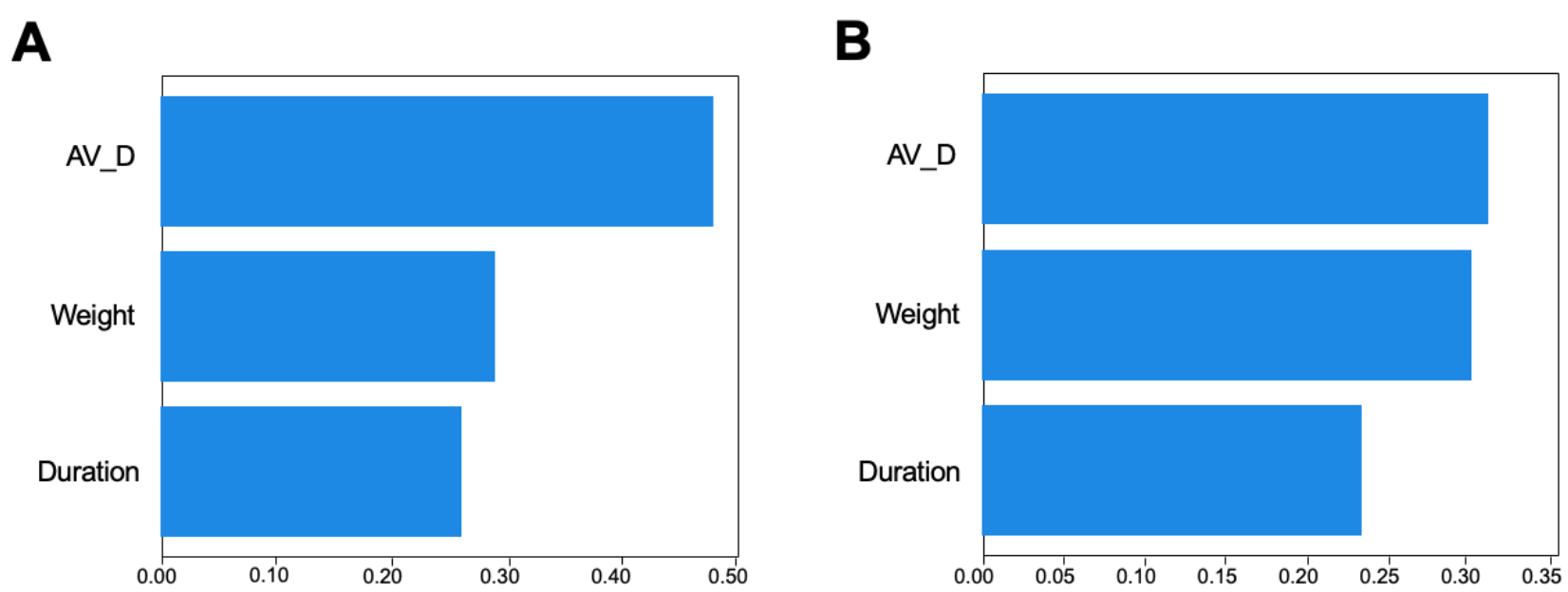

SUPPLEMENTARY FIGURE S2. Variable-importance scores from (A) boosted-tree and (B) bagged-tree models for the absolute difference between paired bevacizumab measurements (AV_D), body weight, and treatment duration in cycles, with the absolute difference between paired vascular endothelial growth factor measurements (VEGF_D) as the target variable. The importance scores are descriptive, not independent of model, and are not indicative of statistical significance.

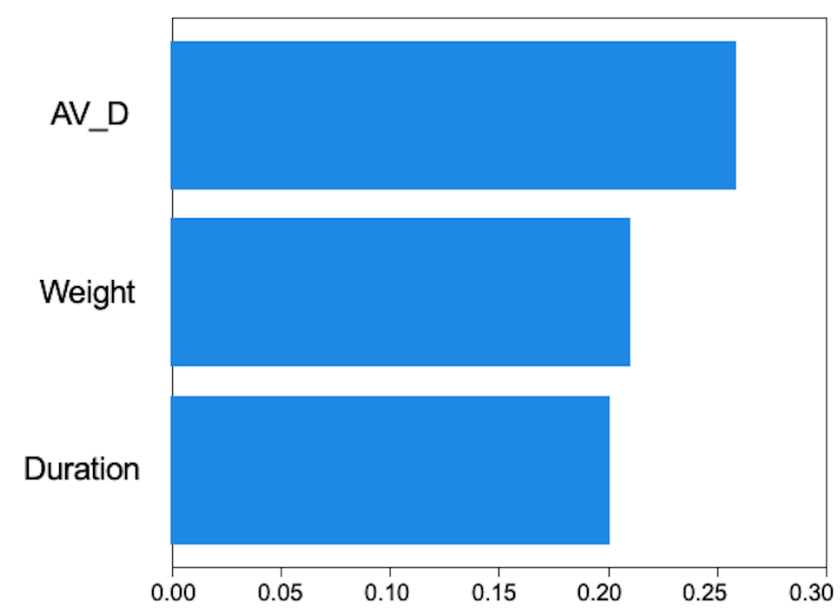

SUPPLEMENTARY FIGURE S3. Variable-importance scores from the complementary exploratory artificial neural network analysis for the absolute difference between the paired bevacizumab measurements (AV_D), body weight, and treatment duration, with the absolute difference between paired vascular endothelial growth factor measurements (VEGF_D) as the target variable. The ANN was fitted to the same cohort as the tree-based models; its output does not constitute independent validation.

Formatted: Space Before: 6 pt, After: 12 pt

Formatted: Font: 11 pt
